# The Effect of Homogenization Heat Treatment on 316L Stainless Steel Cast Billet

**DOI:** 10.3390/ma17010232

**Published:** 2023-12-31

**Authors:** Hung-Yang Chu, Ren-Kae Shiue, Sheng-Yuan Cheng

**Affiliations:** 1 Department of Materials Science and Engineering, National Taiwan University, Taipei 10617, Taiwan; r11527008@ntu.edu.tw; 2 Stainless Steel Business Group, Walsin Lihwa Co., Tainan City 73743, Taiwan; shengyuan_cheng@walsin.com

**Keywords:** homogenization, stainless steel, cast billet, wire drawing, δ-ferrite, sigma phase

## Abstract

This investigation aims to analyze the effect of homogenization heat treatment at 1240 °C for 2 and 6 h on the hardness, distribution, morphology, and chemical composition of the δ-ferrite and sigma phases in 316L stainless steel cast billet. A field emission scanning electron microscope, combined with electron back-scattered diffraction, a field emission electron probe microanalyzer with a wavelength dispersive spectrometer, and a Vickers microhardness tester are applied to identify various phase evolutions in the cast billet. The morphology of the δ-ferrite and sigma phases in the austenite matrix of the 316L cast billet are strongly related to the subsequent hot and cold wire drawings. The homogenization heat treatment is expected to provide a driving force to form spheroid interdendritic δ-ferrite and to minimize the amount of the brittle sigma intermetallic compound in the austenite matrix. The homogenization heat treatment at 1240 °C effectively spheroidized all δ-ferrites into blunt ones in the cast billet. The transformation of δ-ferrite into sigma is dominated by temperature and cooling rate. The fast air cooling after homogenization between 1240 and 850 °C retards the precipitation of the sigma in the δ-ferrite. There are two δ-ferrite transformation mechanisms in this experiment. The direct transformation of the δ-ferrite into sigma is observed in the as-cast 316L stainless steel billet. In contrast, the eutectoid transformation of the δ-ferrite into the sigma and austenite dominates the 316L cast billet homogenized at 1240 °C, with a slow furnace cooling rate.

## 1. Introduction

Chromium-nickel austenitic stainless steels are among the world’s most widely applied engineering alloys due to their excellent formability, weldability, and corrosion resistance [1,2,3]. Due to its high ductility and strength, excellent corrosion resistance, and low cost, 316L stainless steel is comprehensively used in various applications, e.g., medical supplies, fossil and nuclear power plants, petroleum refining industries, and chemical appliances [1,4,5,6]. The 316L stainless steel wire produced from the cast 316L stainless billet experiences a series of hot and cold drawings [7,8]. The formability of the cast billet becomes an important issue in the subsequent manufacturing processes [9,10,11,12].

The 316L stainless steel cast billet contains Mo. It has been reported that a high content of Mo enhances the formation of Mo-rich intermetallics, such as sigma and chi phases, degrading the formability of the cast billet and inducing cracking of the cast billet in subsequent hot/cold wire drawings [13,14]. Therefore, homogenization heat treatment of the cast 316L billet is required before hot drawing [13,15,16,17,18]. The distribution and morphology of δ-ferrite and sigma phases in the austenite matrix of the 316L cast billet are strongly related to the subsequent hot and cold wire drawing of the cast billet. The homogenization heat treatment is expected to provide a driving force to break up the interdendritic δ-ferrite and promote the formation of spheroidal δ-ferrite in the austenite matrix. Because non-uniformly distributed interdendritic flaky δ-ferrite in the 316L stainless steel cast billet deteriorates its formability, a uniformly distributed spheroidal δ-ferrite in the 316L cast billet is beneficial to the subsequent hot and cold drawings of the cast billet. 

The sigma phase formation in the 316 austenitic stainless steel during long-term high-temperature exposure has been reported [19]. The brittle sigma phase is detrimental to the creep strength of the 316 stainless steel [20]. Additionally, a large amount of sigma phase in the 316 stainless steel is related to the corrosion failure of the tube [21]. It is concluded that the sigma phase in the steel significantly deteriorates the performance of the austenitic stainless steel [22,23,24]. The formation of the sigma phase in the 316 stainless steel resulting from various processes, e.g., centrifugal casting, welding, heat treatment, and additive manufacturing, has been reported [25,26,27,28]. The amount, morphology, and location of the sigma phase in the 316L cast billet are strongly related to the fracture of the 316L wire in hot and cold drawings of the cast billet. However, a systematic study of the sigma phase formation in the 316L cast billet using modern analytical analyses has yet to be completed.

Thermo-Calc provides the first approximation in the construction of multicomponent phase diagrams. With the advancement of the thermodynamic database, Thermo-Calc has recently been applied in industry [29]. Many researchers combine DSC (differential scanning calorimetry) and Thermo-Calc calculation to relate the phase transformation temperatures of alloy to optimize the heat treatment condition before the hot/cold working of the alloy [30,31,32,33]. Using these methods, the isothermal sections at selected temperatures and phase fractions upon cooling cycles can be readily achieved with acceptable accuracy [34]. Thermo-Calc is a powerful tool in combination with experimental studies [35,36,37,38].

This investigation aims to clarify the effect of homogenization heat treatment on the amount, distribution, and morphology of δ-ferrite and sigma phase in the 316L stainless steel cast to prevent fracture in the subsequent hot and cold wire drawing processes. A field emission scanning electron microscope (FE-SEM) combined with electron back-scattered diffraction (EBSD), a field emission electron probe microanalyzer (FE-EPMA) with a wavelength dispersive spectrometer (WDS), and a Vickers microhardness tester are applied to identify various phase evolutions in the cast billet. The morphology and distribution of δ-ferrite and sigma phase in the austenite matrix at different locations of the cast billet were evaluated in greater depth. The formation of the sigma phase in the homogenized cast billet was also clarified in the experiment.

## 2. Materials and Experimental Procedures

**Figure 1:**
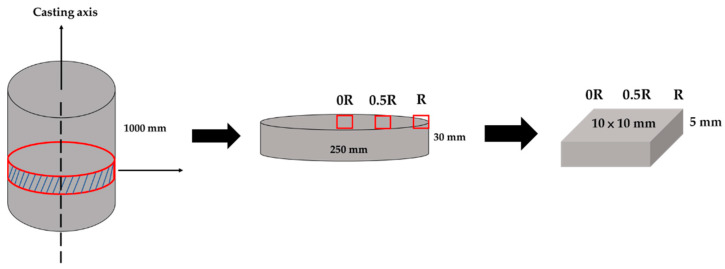
A schematic diagram of the 316L test samples cut from three locations of the disk in the cast billet.

The Walsin Lihwa Corporation prepared a cylinder of the 316L cast billet with a diameter of 250 mm and 1000 mm in length. A disk with a diameter of 250 mm and a thickness of 30 mm was cut from the cast cylinder for experimental use. Figure 1 shows a schematic diagram of the test samples cut from the different locations of the disk. The cast billet was wire-cut and machined to obtain test pieces with a cross-section of approximately 10 mm × 10 mm and a thickness of 5 mm. For comparison, three samples, i.e., from the center, the 1/2 radius, and the radius, were wire cut from the disk of the cast billet. The chemical composition, in wt%, of the 316L cast billet is displayed in Table 1.

**Figure 2:**
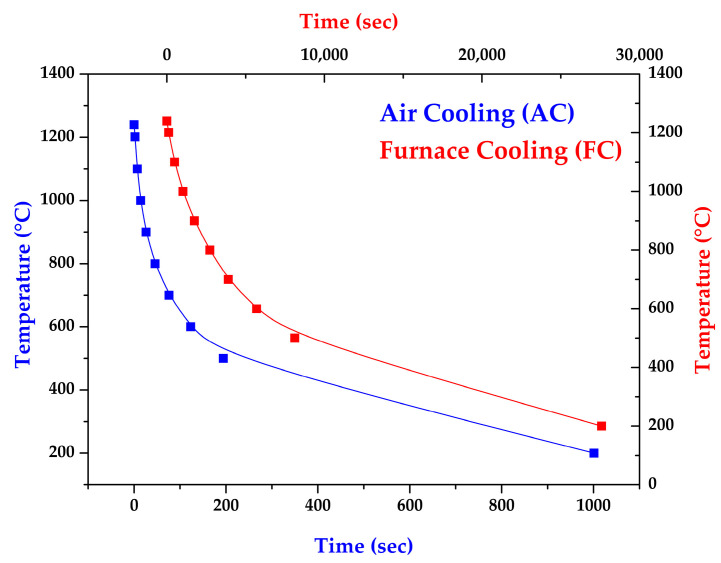
AC and FC cooling curves after homogenization at 1240 °C for 2 and 6 h.

To evaluate the effect of homogenization heat treatment on 316L cast billet, three types of homogenization, namely, no heat treatment, 2 h of heat treatment, and 6 h of heat treatment, were applied for comparison. The test piece was placed into a tubular high-temperature vacuum furnace to avoid oxidation during the heat treatment. A K-type thermocouple was placed on the samples. The homogenization heat treatment was performed at 1240 °C at a vacuum of 5 × 10^−4^ mbar. The heating rate of the high-temperature vacuum furnace was 20 °C/min, and the holding periods were 2 and 6 h, respectively. Two different cooling methods were employed after homogenizing the test pieces at 1240 °C. Figure 2 shows the air cooling (AC) and furnace cooling (FC) curves after homogenization at 1240 °C for 2 and 6 h. Table 2 shows all 316L specimen designations used in the study. C, 0.5R, and R stand for different locations of the specimens taken from the 316L stainless steel cast billet. The 2 h and 6 h time frames represent the homogenization time at 1240 °C. The experiment consists of two cooling rates: AC and FC. For example, C-2h-AC identifies the central part of the cast billet, homogenized at 1240 °C for 2 h, with subsequent air cooling. 

Before the inspection, all test pieces were mounted and underwent a standard metallographic procedure. Each mounted specimen was ground with 400 grit SiC sandpaper, and it was subsequently polished using diamond suspensions with the diamond sizes of 9, 3, 1, and 0.1 μm, respectively. A dilute SiO_2_ suspension was finally used to perform slight chemical etching to relieve the surface stress before the EBSD analysis and optical microscope observation. All surfaces perpendicular to the casting axis shown in Figure 1 were analyzed in the experiment. An FE-SEM (JEOL JSM-7800F Prime, Tokyo, Japan) with an EBSD was applied to perform phase identification and crystallographic analyses. An FE-EPMA (JEOL JXA-8530F Plus, Tokyo, Japan) combined with a WDS was used to perform quantitative chemical analyses of selected positions and mappings of the test pieces. Finally, a Vickers microhardness tester (Mitutoyo HM, Tokyo, Japan) with a load of 10 g and a duration time of 15 s was used to measure the difference in hardness for various phases. 

## 3. Results

**Figure 3:**
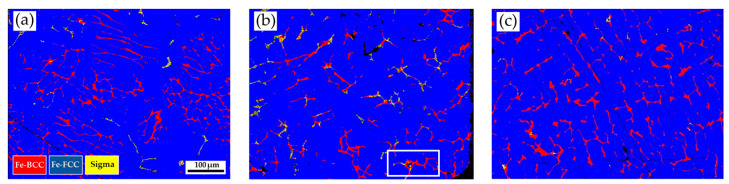
EBSD phase maps of the as-cast 316L stainless steel at different locations: (**a**) center, (**b**) 0.5 R, (**c**) R.

Figure 3 shows the EBSD phase maps of the as-cast 316L stainless steel billet at different locations. The step size used in Figure 3 was 1.3 μm. The cast structure exhibits a dendritic morphology, and the interdendritic δ-ferrite and sigma phases are widely observed in Figure 3. The as-cast billet possesses three phases, including δ-ferrite, sigma, and austenite. The microstructure of the three locations is quite different due to different cooling rates during casting. In Figure 3a, fragmented δ-ferrite (red) is observed in the austenite (blue) matrix in the center of the cast billet. In Figure 3b, the flaky δ-ferrite and sigma phase distributions are still quite uneven at 0.5 R. The distribution of flaky δ-ferrite and sigma phases is improved at the location of R, as displayed in Figure 3c. In Figure 3c, the amount of sigma phase is significantly decreased, and the flaky δ-ferrite becomes blunt at location R. Different microstructures of the cast billet at different locations are expected to result in different responses in the subsequent hot and cold drawings of the cast billet. A homogenization treatment of the cast billet is required before rolling.

**Figure 4:**
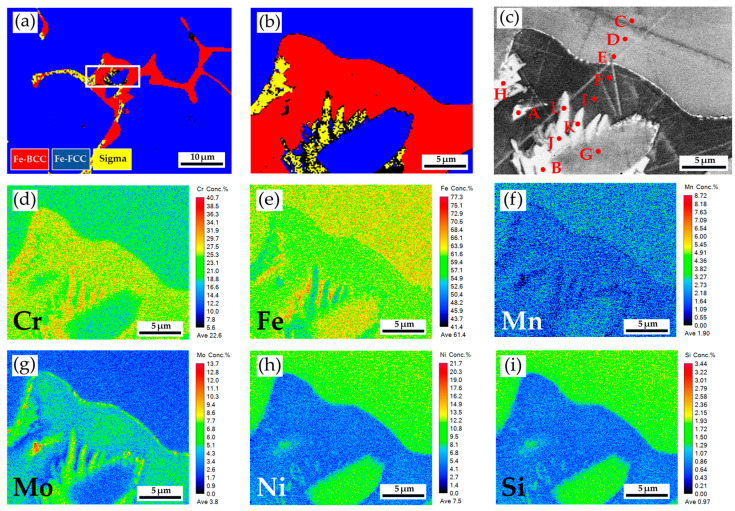
The as-cast 316L stainless steel billet at 0.5R: (**a**,**b**) EBSD phase maps in the area selected in Figure 3b; (**c**) EPMA BEI and quantitative chemical analyses of different locations; (**d**–**i**) EPMA WDS quantitative element mappings of Cr, Fe, Mn, Mo, Ni, and Si.

Figure 4a,b show EBSD phase maps of the as-cast 316L stainless steel billet at 0.5R at higher magnifications. The sigma phase (yellow) is mixed with the δ-ferrite (red) in the austenite matrix (blue). The EBSD phase map in Figure 4b corresponds to the EPMA BEI displayed in Figure 4c. The EPMA WDS quantitative chemical analysis sites at different locations are shown in Figure 4c and Table 3. In Table 3, the carbon concentration in the quantitative chemical analysis of the EPMA/WDS is slightly higher than its actual value due to contamination of the diffusion pump oil in the chamber of the EPMA/WDS. According to Table 3, concentrations of Cr, Mo, and Ni are different in the three phases of the cast billet. The austenite is alloyed with high Ni (>10 at%), low Mo (<3 at%) and Cr (<20 at%) concentrations, as indicated by C, D, E, and G in Figure 4c. In contrast, the δ-ferrite and sigma phases are alloyed with a low concentration of Ni (<5 at%) and a high concentration of Cr (>25 at%), as indicated by B, F, and H~L in Figure 4c. The Cr concentrations of the δ-ferrite and sigma phases are similar, between approximately 25 and 29 at%. However, the Mo concentration in the sigma phase is higher than in the δ-ferrite, as displayed in Table 4. It has been reported that the presence of Mo is attributed to the formation of the sigma phase in the stainless steel [29]. This is consistent with the experimental results.

**Figure 5:**
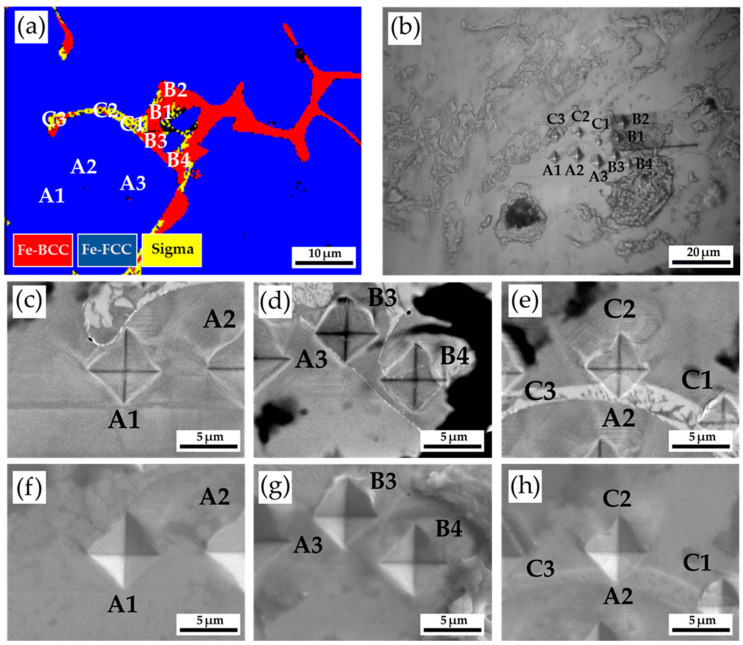
The Vickers hardness measurements of the as-cast 316L stainless steel billet at 0.5 R: (**a**) EBSD phase map in the selected area in Figure 3b; (**b**) the corresponding optical microscope image and Vickers hardness dents of (**a**); the Vickers hardness dents of EPMA (**c**–**e**) BEIs and (**f**–**h**) SEIs.

Figure 5 and Table 4 show the Vickers hardness measurement results of the as-cast 316L stainless steel billet at 0.5 R. The EBSD phase map in Figure 5a corresponds to the optical microscope image showing Vickers hardness dents in Figure 5b. The austenite exhibits the lowest hardness below 200 Hv, as illustrated by A1~A3. The hardness of δ-ferrite, above 200 Hv, is greater than that of austenite, as shown by B1~B4. The sigma phase shows the highest hardness among all phases. However, a considerable variation is observed in the hardness measurement of the sigma phase, as shown in C1~C3. The hardness values of the sigma phase are between 314 and 487. Because the size of the sigma phase is smaller than that of Vickers hardness dent under the applied load of 10 g, both sigma and δ-ferrite are covered by the hardness dent, as shown in Figure 5e. In Figure 5e, the hardness dent at location C1 covers the most significant fraction of the white sigma phase, and it possesses the highest Vickers hardness of 487. It is confirmed that the sigma phase has the highest hardness in the cast billet.

**Figure 6:**
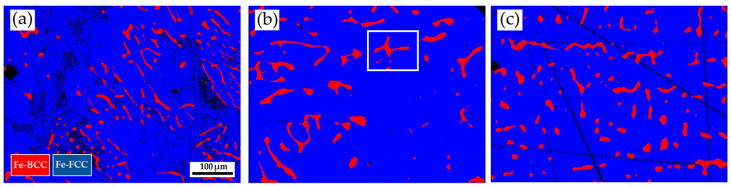
EBSD phase maps of the 316L stainless steel cast billet after homogenization at 1240 °C for 2 h with air cooling at different locations: (**a**) C-2h-AC; (**b**) 0.5 R-2h-AC; (**c**) R-2h-AC.

Figure 6 shows the EBSD phase maps of the 316L stainless steel cast billet after homogenization at 1240 °C for 2 h and air cooling at three locations: the center, 0.5 R, and R. The step size used in Figure 6 is 1.3 μm. The sigma phase has almost disappeared from the entire cast billet. The blunting of the flaky δ-ferrite is widely observed at three locations of the cast billet. However, the spheroidization of the δ-ferrite at the center and 0.5 R is still less prominent than that at R. The homogenization heat treatment effectively improves the flaky δ-ferrite into a blunt sample at all locations. The homogenization heat treatment shows the best effect of spheroidizing δ-ferrite at the location R of the cast billet. It is worth mentioning that the fast air cooling of the cast billet after treatment at 1240 °C for 2 h after homogenization heat treatment is beneficial to avoid the formation of the sigma phase in the entire 316L cast billet.

**Figure 7:**
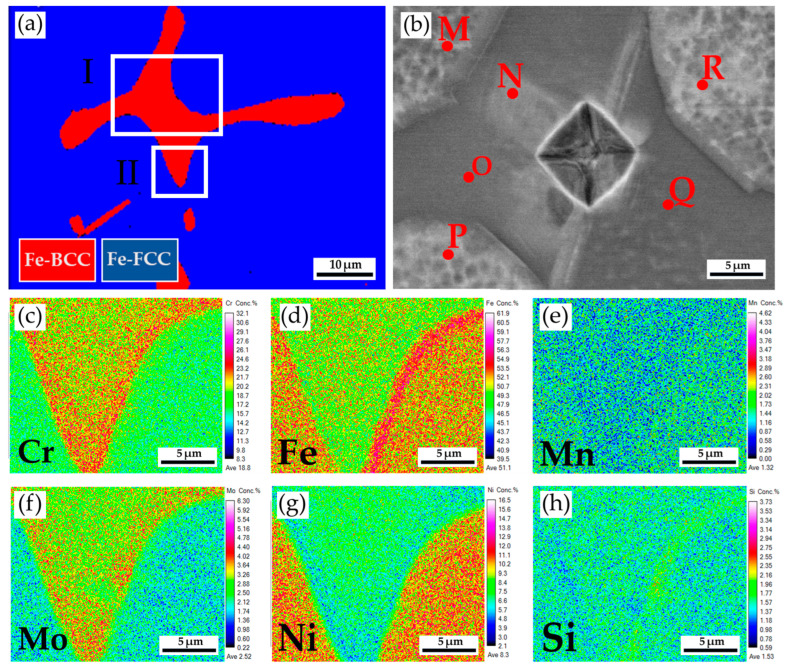
The cast 316L stainless steel billet at 0.5 R after homogenization at 1240 °C for 2 h, with air cooling: (**a**) EBSD phase map in the area selected in Figure 6b; (**b**) EPMA BEI and quantitative chemical analyses of different locations in area I of (**a**); (**c**–**h**) EPMA WDS quantitative element mappings of Cr, Fe, Mn, Mo, Ni, and Si in the area II of (**a**).

Figure 7a displays the EBSD phase maps of the 316L stainless steel cast billet, at a higher magnification, after homogenization at 1240 °C for 2 h and air cooling at 0.5 R. The sigma phase disappeared from the figure due to the fast air cooling after homogenization. The area I of the EBSD phase map in Figure 7a corresponds to the EPMA BEI displayed in Figure 7b. The EPMA WDS quantitative chemical analysis sites at different locations are shown in Figure 7b and Table 5. Similar to the results above, the austenite is alloyed with high Ni (>10 at%), low Mo (<3 at%), and Cr (<20 at%) concentrations, as indicated by M, P, and R in Figure 7b. On the other hand, the δ-ferrite is alloyed with a low concentration of Ni (<7.4 at%) and a high concentration of Cr (>21.9 at%), as indicated by N, O, and Q in Figure 7b. The Mo concentration in the δ-ferrite is slightly higher than in the austenite, as shown in Table 5. Figure 7c–h shows EPMA WDS quantitative element mappings of Cr, Fe, Mn, Mo, Ni, and Si, consistent with the results shown in Table 5. It is noted that the Cr and Mo are enriched in the δ-ferrite close to the interface between the δ-ferrite and austenite. The δ-ferrite alloyed with high Cr and Mo concentrations favors the sigma phase formation, which will be discussed later. Figure 3 and Figure 6 show that the microstructure at 0.5 R (Figure 3b and Figure 6b) is intermediated between those at the center and R (Figure 3a,c and Figure 6a,c). The following analyses are focused on the microstructures of 0.5 R cast billet. Additionally, the rapid air cooling of the 316L cast billet after homogenization at 1240 °C for 2h results in a microstructure free of the sigma phase. Because the objective of avoiding the brittle sigma phase is achieved, the rapid air cooling of the 316L cast billet after homogenization at 1240 °C for 6 h is not performed in the experiment.

**Figure 8:**
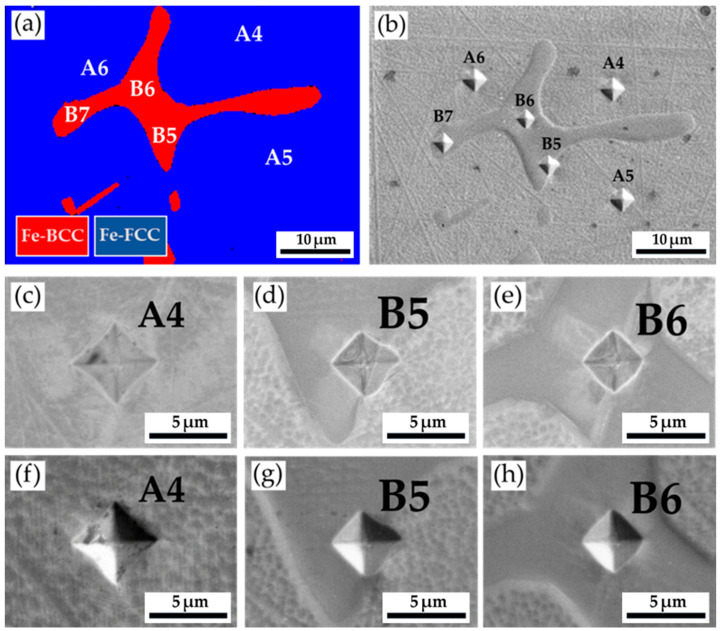
The Vickers hardness measurements of the as-cast 316L stainless steel billet at 0.5 R after homogenization at 1240 °C for 2 h, with air cooling: (**a**) EBSD phase map in the area selected in Figure 6b, (**b**) the corresponding EPMA SEI, with the Vickers hardness dents of (**a**); Vickers hardness dents of EPMA (**c**–**e**) BEIs and (**f**–**h**) SEIs.

Table 6 and Figure 8 display the Vickers hardness measurements of the as-cast 316L stainless steel billet at 0.5 R after homogenization at 1240 °C for 2 h, with air cooling. The EBSD phase map in Figure 8a corresponds to the EPMA SEI, with Vickers hardness dents, in Figure 8b. The hardness of austenite is below 200 Hv. The hardness of δ-ferrite is higher than 200 Hv, i.e., between 200 and 251. The homogenization heat treatment does not significantly change the hardness of austenite and δ-ferrite as compared to the results in Table 4 and Table 6.

**Figure 9:**
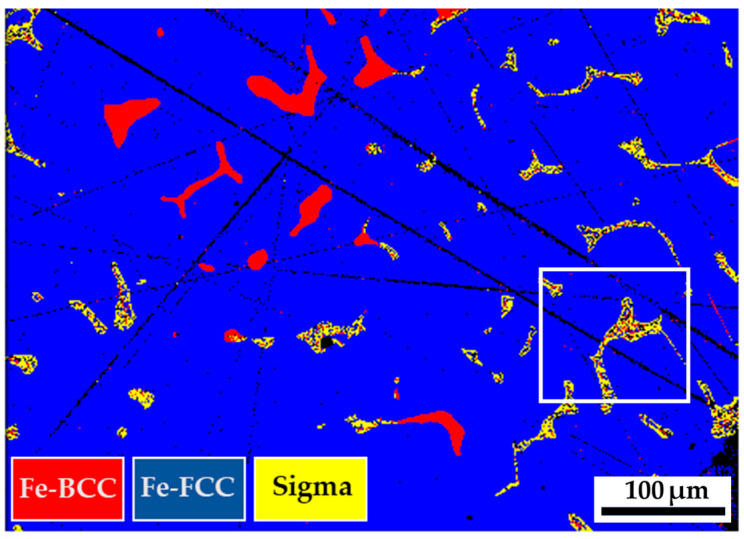
The EBSD phase map of the 316L stainless steel cast billet after homogenization at 1240 °C for 2 h, with furnace cooling, at location 0.5 R.

**Figure 10:**
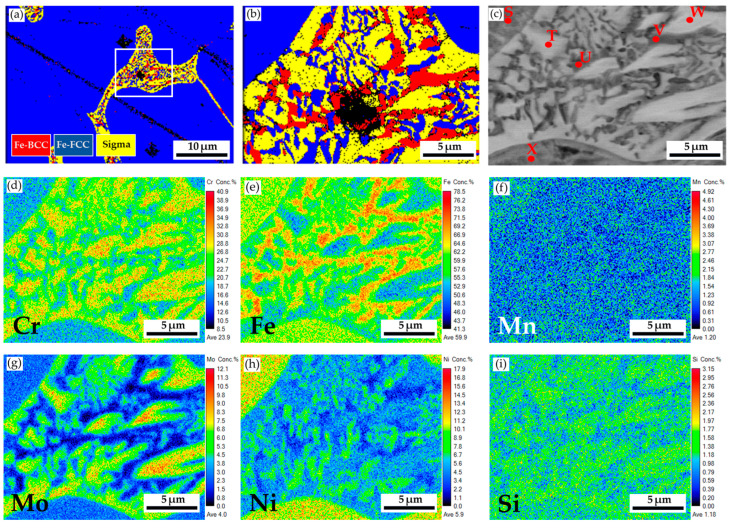
The cast 316L stainless steel billet at 0.5 R after homogenization at 1240 °C for 2 h, with furnace cooling: (**a**,**b**) EBSD phase maps of areas selected in Figure 9; (**c**) EPMA BEI and quantitative chemical analyses of different locations; (**d**–**i**) EPMA WDS quantitative element mappings of Cr, Fe, Mn, Mo, Ni, and Si.

Figure 9 shows the EBSD phase map of the 316L stainless steel cast billet after homogenization at 1240 °C for 2 h and furnace cooling at location 0.5 R. The blunting of the flaky δ-ferrite shown in Figure 9 is not apparent under the slow furnace cooling condition compared to that observed under the fast air cooling condition, as shown in Figure 6b. A slow furnace cooling rate also forms a vast amount of sigma phase in the cast billet. According to Figure 9, many δ-ferrite islands are partially transformed into the sigma phase, and only a few blocky δ-ferrites are left in the austenite matrix. Figure 10a is the EBSD phase map of the area selected in Figure 9, and Figure 10b is the EBSD phase map at a higher magnification than that used in Figure 10a. Figure 10b shows a mixture of blue austenite, red δ-ferrite, and yellow sigma phases in the original δ-ferrite island. The original δ-ferrite has partially decomposed into austenite and sigma phases. Figure 10c and Table 7 display the EPMA BEI and quantitative chemical analyses of different locations. The austenite is alloyed with high Ni (>10 at%) and low Mo (<3 at%) and Cr (<19 at%) concentrations, as indicated by S and X in Figure 10c. Both δ-ferrite and sigma phases exhibit low Ni (<5 at%) and high Cr (>20 at%) concentrations, as shown by T, U, V, and W in Figure 10c. However, the sigma phase is alloyed with the highest Cr (>26 at%) and Mo (>7 at%) concentrations, as displayed in Table 7. Figure 10d–i display the EPMA WDS quantitative element mappings of Cr, Fe, Mn, Mo, Ni, and Si at the identical location shown in Figure 10c. The white sigma phase shown in Figure 10c coincides with the Cr and Mo mappings in Figure 10d,g. Combining high Cr and Mo concentrations and slow furnace cooling favors sigma phase formation.

**Figure 11:**
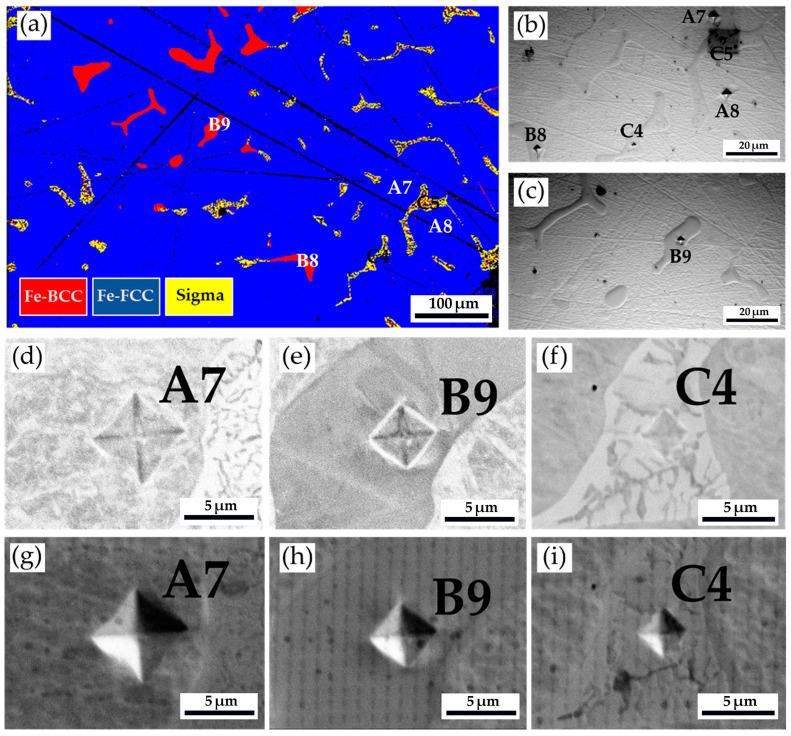
The Vickers hardness measurements of the as-cast 316L stainless steel billet at 0.5 R after homogenization at 1240 °C for 2 h, with furnace cooling: (**a**) EBSD phase map in Figure 9; (**b**,**c**) the corresponding optical metallographs with Vickers hardness dents of (**a**); Vickers hardness dents of EPMA (**d**–**f**) BEIs and (**g**–**i**) SEIs.

Figure 11 and Table 8 show the Vickers hardness measurements of the as-cast 316L stainless steel billet at 0.5 R after homogenization at 1240 °C for 2 h, with slow furnace cooling. The corresponding EPMA SEIs/BEIs with the Vickers hardness dents of Figure 11a are also included to validate the locations of different phases in the hardness test. The hardness of austenite is quite soft, below 200 Hv, which is similar to the results above. In contrast, the hardness of δ-ferrite is significantly increased up to 402 Hv, as compared with that in Table 4 and Table 6. Because the δ-ferrite hardness is seldom above 300 Hv, the preliminary transformation could occur in the δ-ferrite grains. The sigma phase exhibits the highest Vickers hardness of 741. The high hardness of the sigma phase is detrimental to the subsequent hot/cold forging of the 316L cast billet. It has better to remove the sigma phase before the subsequent forging process. 

**Figure 12:**
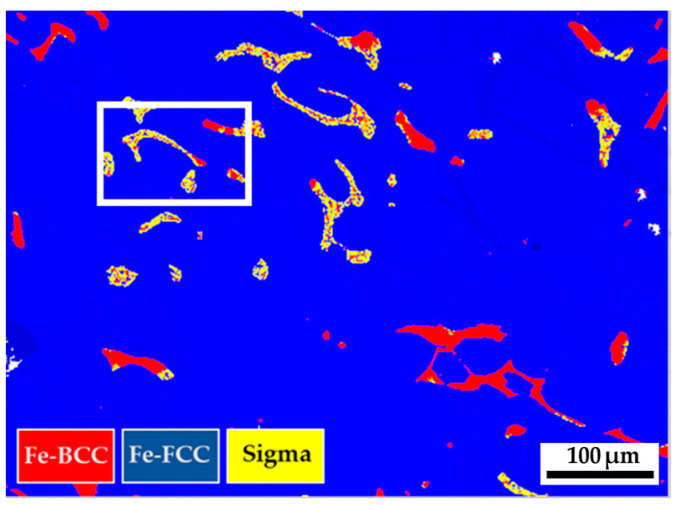
The EBSD phase map of the 316L stainless steel cast billet after homogenization at 1240 °C for 6 h, with furnace cooling, at 0.5 R.

**Figure 13:**
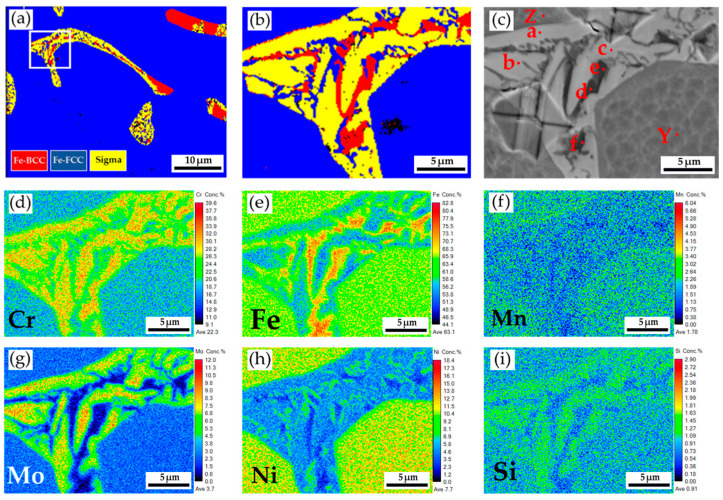
The cast 316L stainless steel billet at 0.5 R after homogenization at 1240 °C for 6 h, with furnace cooling: (**a**,**b**) EBSD phase maps in areas selected in Figure 12; (**c**) EPMA BEI and quantitative chemical analyses of different locations; (**d**–**i**) EPMA WDS quantitative element mappings of Cr, Fe, Mn, Mo, Ni, and Si.

Figure 12 displays the EBSD phase maps of the 316L stainless steel cast billet after homogenization at 1240 °C for 6 h, with furnace cooling, at location 0.5 R. The microstructure with 6 h homogenization illustrated in Figure 12 is similar to that in Figure 9, with 2 h homogenization. The blunting of the flaky δ-ferrite under slow furnace cooling conditions shown in Figure 9 and Figure 12 is inferior to that shown in Figure 6b, under the fast air cooling conditions. The extension of the homogenization time at 1240 °C from 2 to 6 h has little effect on the microstructure of the 316L cast billet. Figure 13a,b shows the EBSD phase maps of selected areas in Figure 12. The original flaky δ-ferrite islands could transform into a mixture of austenite, sigma, and retained δ-ferrite, as illustrated in Figure 13b. Figure 13c is the corresponding EPMA BEI of Figure 13b, and the quantitative chemical analyses of different locations in Figure 13c are displayed in Table 9. The sigma phase has high Cr and Mo concentrations, indicated by a, b, and c in Figure 13c. The austenite is alloyed with low Cr and high Ni concentrations, as indicated by Y and Z. It is important to note that the δ-ferrite is alloyed with a deficient Mo concentration below 1.0 at%, as denoted by d, e, and f in Figure 13c. The transformation of δ-ferrite into the sigma phase results from the enrichment of the Mo in the sigma phase. The migration of Mo in the original δ-ferrite plays an important role in forming the sigma phase. Figure 13d–i shows the EPMA WDS quantitative element mappings of Cr, Fe, Mn, Mo, Ni, and Si. The sigma phase is rich in Cr and Mo, as demonstrated in Figure 13d,g. The austenite is rich in Ni, as displayed in Figure 13h. They are all consistent with the EPMA analysis results. 

**Figure 14:**
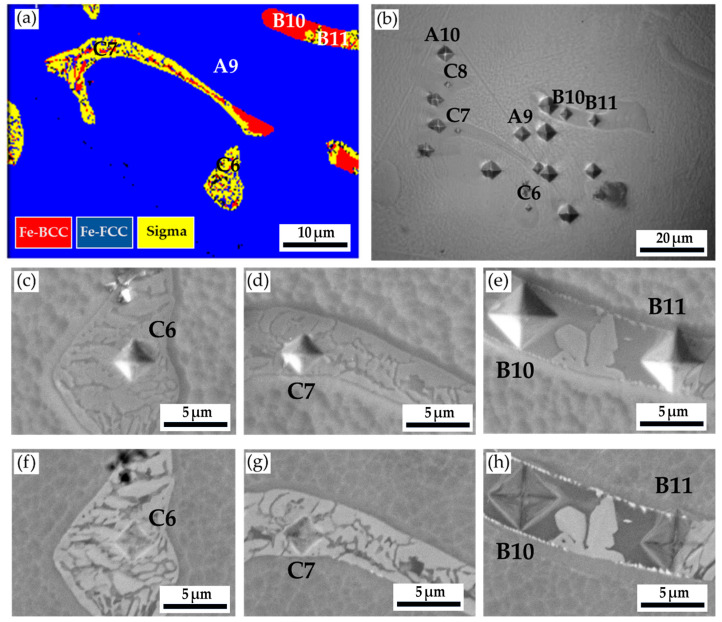
The Vickers hardness measurements of the as-cast 316L stainless steel billet at 0.5 R after homogenization at 1240 °C for 6 h, with furnace cooling: (**a**) EBSD phase map in the area selected in Figure 12; (**b**) the corresponding optical metallograph, with Vickers hardness dents of (**a**); Vickers hardness dents of EPMA (**c**–**e**) SEIs and (**f**–**h**) BEIs.

Figure 14 and Table 10 show the Vickers microhardness test results of the cast 316L stainless steel billet at 0.5 R after homogenization at 1240 °C for 6 h, with slow furnace cooling. Figure 14a shows the EBSD phase map at the area selected in Figure 12, and Figure 14b displays the corresponding optical metallograph, with Vickers hardness dents, of Figure 14a. The Vickers hardness dents of EPMA SEIs (Figure 14c–e) and BEIs (Figure 14f–h) SEIs are also included in the figure. The hardness of austenite is the softest phase, below 200 Hv. The hardness of δ-ferrite is higher than that of austenite. The sigma phase has the highest hardness value of 860 Hv. The presence of high-hardness sigma intermetallics is detrimental to the subsequent forging of the 316L cast billet.

**Figure 15:**
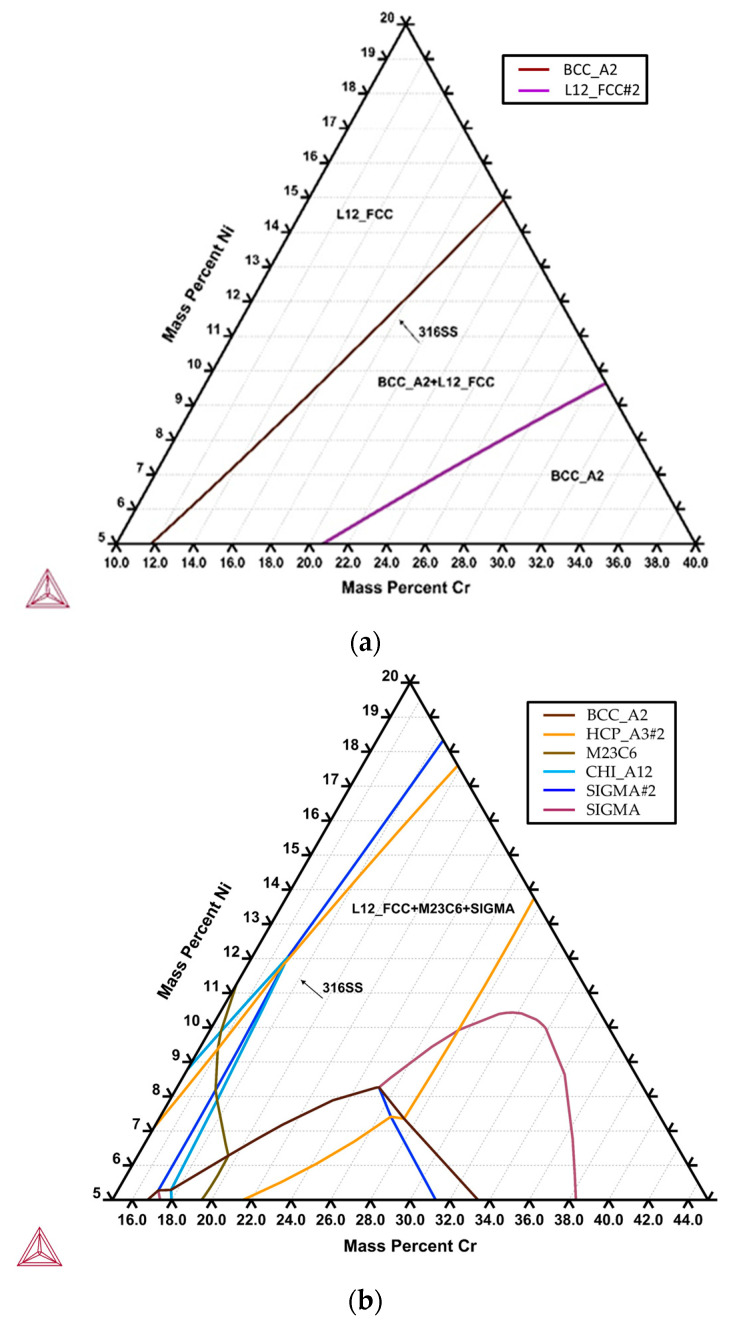
The isothermal sections of the 316L stainless steel at (**a**) 1240 and (**b**) 850 °C were simulated by Thermo-Calc.

Figure 15 shows the isothermal sections of the 316L stainless steel at 1240 and 850 °C, respectively, simulated by Thermo-Calc. In Figure 15a, the chemical composition of the 316L cast billet is in the two-phase region, with δ-ferrite (BCC) and austenite (FCC), consistent with the experimental observation. According to Figure 15b, the equilibrium phases at 850 °C in the 316L stainless steel are FCC austenite, M_23_C_6_ carbide, and the sigma phase. The 316L cast billet is alloyed with a low carbon concentration of 0.02 wt%, so it is reasonable that the M_23_C_6_ carbide is not observed in the experiment. However, the δ-ferrite is not in an equilibrium phase at 850 °C in the isothermal section (Figure 15b). this is not consistent with the experimental observation. The Thermo-Calc simulation deviates from the experimental result. Because the formation of the sigma phase is a kinetic issue, i.e., a rate-dependent process, the simulation based on thermodynamics is not appropriate. The Thermo-Calc simulation is unsuitable, especially for the case of solid-state transformation due to slow diffusion to reach the final equilibrium state.

**Figure 16:**
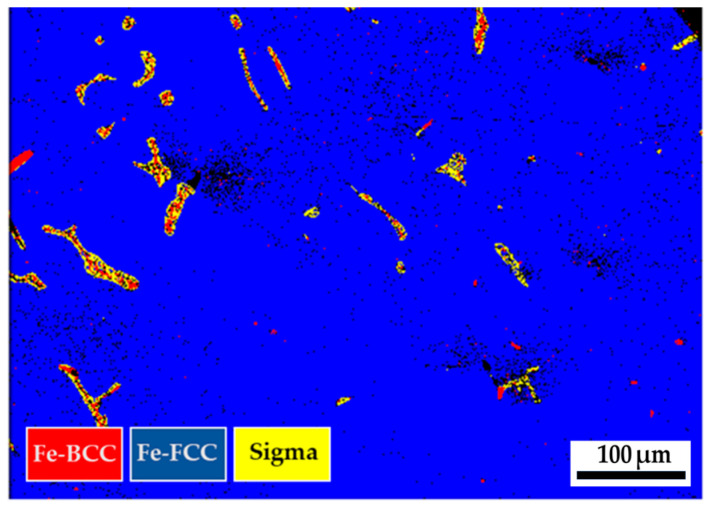
The EBSD phase map of the 316L stainless steel cast billet after homogenization at 1240 °C for 2 h, cooled to 850 °C with a slow furnace, followed by a fast air cooling to room temperature.

Figure 16 shows the EBSD phase map of the 316L stainless steel cast billet after homogenization at 1240 °C for 2 h, cooled to 850 °C with a slow furnace, followed by a fast air cooling to room temperature. The original δ-ferrite islands have partially transformed into the sigma phase, a few austenite particles, and retained δ-ferrite. The microstructure shown in Figure 16 is similar to those shown in Figure 9 and Figure 12. Fast air cooling from 850 °C to room temperature after homogenization heat treatment cannot prohibit sigma phase formation. In contrast, the sigma phase has almost disappeared from the entire cast billet under fast air cooling after the homogenization heat treatment at 1240 °C, as demonstrated in Figure 6. It is deduced that rapid cooling is required, between 850 and 1240 °C, after homogenization treatment to avoid the sigma phase formation in the 316L cast billet.

## 4. Discussion

In Figure 1, the large size of the cylindrical 316L stainless steel cast billet made it impossible to cool uniformly. Different heat transfer rates at the center, 0.5 radii, and radius of the cast billet result in different cooling rates. Additionally, the latent solidification heat is dissipated from the center into the circumference of the cylindrical cast billet. The circumference of the cast billet solidified first due to the fast cooling rate. A dendritic morphology is observed in Figure 3c. In contrast, the cooling rates at the center and 0.5 radii of the cast billet are much slower than those at the circumference of the cast billet. This is demonstrated by a higher volume fraction of the sigma phase in Figure 3a,b. The morphologies of interdendritic δ-ferrite and sigma phases in the center and 0.5 radii of the cast billet are quite different from those at the circumference of the cast billet, as illustrated in Figure 3. 

There are mixtures of δ-ferrite and sigma phases in the as-cast and homogenized 316L stainless steel cast billet. The difference between the δ-ferrite and sigma phases cannot be readily distinguished according to the quantitative chemical analysis results of the two phases. The EBSD crystallographic analysis and the microhardness measurement provide two approaches to distinguish these two phases. However, the Vickers microhardness measurement is an easier method for identifying the presence of the sigma phase in industrial applications, especially for on-site examination. 

The minimum load of the Vickers microhardness test is 10 g, which is applied in the experiment to decrease the size of the hardness dent. The size of the microhardness dent is small enough to support the austenite matrix with a hardness below 200 Hv, and the variation in hardness values for the austenite is quite tiny. In contrast, the variation of the Vickers hardness is increased for the δ-ferrite between 200 and 402 Hv. Traditionally, the hardness of δ-ferrite is below 300 Hv. The partial transformation of δ-ferrite into the sigma phase and the formation of a mixture of both phases at the location of the original interdendritic δ-ferrite results in a higher variation of its hardness. The sigma phase exhibits the highest Vickers microhardness and the greatest variation due to the limited size of the sigma phase. According to the experimental result, the Vickers microhardness values of the sigma phase are between 314 and 860. The size of the microhardness dent may cover both the sigma and δ-ferrite phases. The combined EBSD crystallographic analyses and the Vickers hardness measurements demonstrate the above phenomenon. 

It has been reported that the precipitation of the sigma phase is associated with embrittlement [39,40]. According to Table 1, the Cr equivalent (%Cr + %Mo + 1.5 × %Si + 0.5 × %Nb in wt%) equals 22.18, and the Ni equivalent (%Ni + 30 × %C + 0.5 × %Mn) equals 12.91. Because the Cr _eq_/Ni _eq_ equals 1.71, the solidification of the 316L cast billet in the experiment belongs to the type of FA mode [41,42]. During the transformation of δ-ferrite to austenite, the δ-ferrite is enriched in Cr, Mo, and Si. The δ-ferrite eventually transforms to the austenite and sigma phase in welding the 316L stainless steel [27,42]. The transformation of δ-ferrite to the sigma phase depends on the Cr, Mo, and Si concentrations in the δ-ferrite, and the enrichment process is controlled by the kinetics of the δ-ferrite dissolution [27]. It has also been reported that the precipitation of M_23_C_6_ has been shown to denude the δ-ferrite of Cr and Mo and lower the propensity for sigma formation [27]. Because the average carbon concentration of 316L stainless steel cast billet is below 0.03 wt%, the sigma formation is promoted in the experiment. This is consistent with the experimental observation. 

The sigma phase is observed in the center and 0.5 R of the as-cast billet. However, there is no sigma phase in the R of the cylindrical as-cast billet. The transformation of δ-ferrite into the sigma phase is a diffusion-controlled process dominated by precipitation temperature, dwelling time, and cooling rate [27,41,42,43]. The fast air cooling is associated with a limited time to proceed with the diffusion of Cr and Mo in the δ-ferrite, and it retards the precipitation of the sigma in the δ-ferrite. In contrast, slow cooling rates at the center and 0.5 R of the cast billet rate promote the precipitation of the sigma phase from δ-ferrite in the experiment. It has been reported that there are two mechanisms of sigma precipitation from the interdendritic δ-ferrite, including direct transformation (δ-ferrite → sigma) and eutectoid transformation (δ-ferrite → sigma + austenite) [42]. The direct transformation of the δ-ferrite into sigma is widely observed in the as-cast 316L billet illustrated in Figure 4a,b and Figure 5a. The eutectoid transformation of the δ-ferrite into sigma and austenite dominates the 316L cast billet homogenized at 1240 °C, with slow furnace cooling, in the experiment. This is demonstrated by the mixture of δ-ferrite, sigma, and austenite displayed in Figure 9, Figure 10a,b, Figure 11a, Figure 12, Figure 13a,b, Figure 14a, and Figure 16.

## 5. Conclusions

The effect of homogenization heat treatment on the 316L stainless steel cast billets at 1240 °C for 2 and 6 h has been investigated. Combining FESEM/EBSD, FEEPMA/WDS, and Vickers microhardness analyses provides a novel approach for identifying various phase evolutions in the cast billet. Some important conclusions are summarized below.

1. The homogenization heat treatment at 1240 °C effectively spheroidized all δ-ferrites into blunt samples in the cast billet.
2. The austenite matrix shows the lowest hardness below 200 Hv_0.01_, with minimal variation. In contrast, the variation in the Vickers hardness is increased for the δ-ferrite between 200 and 402 Hv_0.01_ due to the partial transformation of δ-ferrite into the sigma phase. Because of its tiny size in the cast billet, the sigma phase exhibits a Vickers microhardness with a maximum variation between 314 and 860 Hv_0.01_.
3. The transformation of δ-ferrite into sigma is dominated by temperature and cooling rate. The fast air cooling after homogenization between 1240 and 850 °C retards the precipitation of the sigma in the δ-ferrite.
4. The direct transformation of the δ-ferrite into sigma is observed in the as-cast 316L billet. In contrast, the eutectoid transformation of the δ-ferrite into sigma and austenite dominates the 316L cast billet homogenized at 1240 °C, with a slow furnace cooling rate.

## Figures and Tables

**Table 1:** The chemical composition, in wt%, of the 316L cast billet.

Element	C	Co	Cr	Cu	Mn	Mo	N	Ni	P	S	Si	V	Fe
wt%	0.02	0.13	18.15	0.12	1.85	2.62	0.05	11.38	0.03	0.01	0.94	0.10	Bal.

**Table 2:** The 316L specimen designation used in the study.

Symbol	Heat Treatment Procedure	Location
C	as cast	Center
0.5 R	as cast	0.5 R
R	as cast	R
C-2 h-AC	1240 °C for 2 h, air cooling	Center
0.5 R-2 h-AC	1240 °C for 2 h, air cooling	0.5 R
R-2 h-AC	1240 °C for 2 h, air cooling	R
0.5 R-2 h-FC	1240 °C for 2 h, furnace cooling	0.5 R
0.5 R-2 h-850 °C-AC	1240 °C for 2 h, furnace cooled to 850 °C, and fast air cooling	0.5 R
0.5 R-6 h-FC	1240 °C for 6 h, furnace cooling	0.5 R

**Table 3:** EPMA WDS quantitative chemical analyses in at% of A~L in Figure 4c.

Element/at%	C	Cr	Fe	Mn	Mo	Ni	O	P	Si	Si	Phase
A	0.9	21.1	56.7	1.9	10.4	7.3	0.0	0.3	0.1	1.3	---
B	0.8	28.2	56.1	1.5	7.5	4.6	0.0	0.1	0.0	1.2	sigma
C	0.2	17.7	65.4	1.9	2.2	11.8	0.0	0.0	0.0	0.8	austenite
D	1.2	18.1	64.2	2.0	2.4	11.2	0.0	0.0	0.0	0.9	austenite
E	1.1	18.7	64.2	2.0	2.6	10.5	0.0	0.0	0.0	0.9	austenite
F	0.9	25.8	60.6	1.6	5.1	4.8	0.0	0.1	0.0	1.1	δ-ferrite
G	0.8	19.1	64.4	1.9	2.7	10.2	0.0	0.0	0.0	0.9	austenite
H	1.1	27.7	57.7	1.7	6.2	4.3	0.0	0.1	0.0	1.2	sigma
I	0.8	26.2	61.2	1.6	4.5	4.6	0.0	0.1	0.0	1.0	δ-ferrite
J	2.1	28.2	54.9	1.6	7.1	4.6	0.0	0.1	0.2	1.2	sigma
K	2.1	26.7	56.9	1.6	6.4	4.9	0.0	0.1	0.1	1.2	sigma
L	2.1	27.9	54.7	1.6	7.5	4.5	0.0	0.2	0.2	1.3	sigma

**Table 4:** Vickers hardness measurements displayed in Figure 5.

Location	A1	A2	A3	B1	B2	B3	B4	C1	C2	C3
Hv (10 g)	196	171	184	200	214	251	260	487	314	328

**Table 5:** EPMA WDS quantitative chemical analyses in at% of M~R in Figure 7b.

Element /at%	C	Cr	Fe	Mn	Mo	Ni	O	P	Si	Si	Phase
M	0.1	18.8	65.4	1.5	2.4	10.9	0.0	0.0	0.1	0.8	austenite
N	0.2	23.7	62.6	1.2	4.1	7.1	0.0	0.1	0.1	0.9	δ-ferrite
O	0.2	23.6	62.6	1.4	4.1	6.9	0.0	0.1	0.1	1.0	δ-ferrite
P	0.1	18.3	65.8	1.4	2.4	11.1	0.0	0.0	0.1	0.8	austenite
Q	0.2	21.9	64.1	1.3	3.9	7.4	0.0	0.1	0.1	1.0	δ-ferrite
R	0.2	18.1	65.6	1.5	2.5	11.1	0.0	0.0	0.1	0.9	austenite

**Table 6:** Vickers hardness measurements displayed in Figure 7.

Location	A4	A5	A6	B5	B6	B7
Hv (10 g)	196	171	184	200	214	251

**Table 7:** EPMA WDS quantitative chemical analyses in at% of S~X in Figure 10c.

Element /at%	C	Cr	Fe	Mn	Mo	Ni	O	P	Si	Si	Phase
S	0.1	18.6	65.2	1.3	2.6	11.2	0.0	0.0	0.1	0.9	austenite
T	0.1	28.2	56.7	1.2	7.6	4.7	0.0	0.1	0.2	1.2	sigma
U	0.1	21.1	73.7	0.8	1.0	2.6	0.0	0.0	0.0	0.7	δ-ferrite
V	0.2	20.9	74.1	0.8	0.8	2.5	0.0	0.0	0.0	0.7	δ-ferrite
W	0.1	26.2	58.8	1.2	7.7	4.5	0.0	0.1	0.2	1.2	sigma
X	0.1	17.3	67	1.3	2.6	10.7	0.0	0.0	0.1	0.9	austenite

**Table 8:** Vickers hardness measurements displayed in Figure 11.

Location	A7	A8	B8	B9	C4	C5
Hv (10 g)	173	179	363	402	691	741

**Table 9:** EPMA WDS quantitative chemical analyses in at% of Y~f in Figure 13c.

Element /at%	C	Cr	Fe	Mn	Mo	Ni	O	P	Si	Si	Phase
Y	0.5	18.3	64.5	1.9	2.4	11.3	0.0	0.0	0.1	1.0	austenite
Z	0.5	18.6	64.4	1.9	2.5	11.0	0.0	0.0	0.1	1.0	austenite
a	0.5	29.0	55.6	1.6	7.4	4.5	0.0	0.1	0.2	1.1	sigma
b	0.5	28.6	55.2	1.7	7.6	4.9	0.0	0.1	0.2	1.2	sigma
c	0.6	28.6	55.9	1.7	6.8	5.0	0.0	0.1	0.1	1.2	sigma
d	0.5	20.9	71.7	1.2	0.8	4.2	0.0	0.0	0.0	0.7	δ-ferrite
e	0.5	23.6	69.6	1.1	1.0	3.2	0.0	0.0	0.0	1.0	δ-ferrite
f	0.6	21.2	73.5	1.0	0.5	2.6	0.0	0.0	0.0	0.6	δ-ferrite

**Table 10:** Vickers hardness measurements displayed in Figure 14.

Location	A9	A10	B10	B11	C6	C7	C8
Hv (10 g)	173	142	223	287	646	860	605

## Data Availability

Data are contained within the article.
